# Draft Genome Sequences of Eight Type Strains of the Genus *Demequina*

**DOI:** 10.1128/genomeA.00281-15

**Published:** 2015-04-16

**Authors:** Moriyuki Hamada, Natsuko Ichikawa, Akio Oguchi, Hisayuki Komaki, Tomohiko Tamura, Nobuyuki Fujita

**Affiliations:** a Biological Resource Center, National Institute of Technology and Evaluation (NBRC), Chiba, Japan; b NBRC, Tokyo, Japan

## Abstract

Here, we report the draft genome sequences of the type strains of *Demequina aestuarii*, *Demequina aurantiaca*, *Demequina flava*, *Demequina globuliformis*, *Demequina lutea*, *Demequina oxidasica*, *Demequina salsinemoris*, and *Demequina sediminicola*. The genome sequences presented here will facilitate taxonomical, ecological, and functional studies of members of the genus *Demequina*.

## GENOME ANNOUNCEMENT

The genus *Demequina*, which belongs to the family *Demequinaceae* within the suborder *Micrococcineae*, is composed of Gram stain-positive, rod-shaped, and non-endospore-forming actinobacteria (1, 2). At the time of writing, this genus contains 8 species with validly published names: *Demequina aestuarii* (1), *Demequina aurantiaca*, *Demequina globuliformis*, *Demequina oxidasica* (2), *Demequina flava*, *Demequina sediminicola* (3), *Demequina lutea* (4), and *Demequina salsinemoris* (5). So far, members of the genus were mainly isolated from samples of marine origin, such as tidal flat and sea sediments. The genus *Demequina* is characterized by the presence of a discriminative menaquinone, namely, demethylmenaquinone DMK-9(H_4_). Furthermore, the peptidoglycan type of this genus is of A4β type with ornithine as the diagnostic diamino acid (6), and this feature distinguishes the genus *Demequina* from the closely related genus *Lysinimicrobium* within the family *Demequinaceae* (7). To reveal genomic features of the genus *Demequina*, we performed whole-genome shotgun sequencing of all type strains of the genus.

Genomic DNAs of *D. aestuarii* NBRC 106260^T^, *D. aurantiaca* NBRC 106265^T^, *D. flava* NBRC 105854^T^, *D. globuliformis* NBRC 106266^T^, *D. lutea* NBRC 106155^T^, *D. oxidasica* NBRC 106264^T^, *D. salsinemoris* NBRC 105323^T^, and *D. sediminicola* NBRC 105855^T^ were extracted and purified from liquid-dried cells in ampules provided from the NBRC culture collection using the EZ1 DNA tissue kit and EZ1 advanced instruments (Qiagen). The whole genomes of these strains were analyzed by using paired-end sequencing with MiSeq (Illumina). These reads were assembled using the Newbler v2.6 software and subsequently finished using the GenoFinisher software (8). The results of the sequencing are summarized in Table 1.

**TABLE 1:** Summary of genome sequencing in the present study

Organism	Read (Mb)	Fold coverage	No. of scaffolds	Genome size (bp)	G+C content (%)	Accession no.
*D. aestuarii* NBRC 106260^T^	291	104	3	2,790,392	69.1	BBRD00000000
*D. aurantiaca* NBRC 106265^T^	265	103	4	2,565,694	64.6	BBRF00000000
*D. flava* NBRC 105854^T^	215	84	3	2,546,965	64.7	BBRA00000000
*D. globuliformis* NBRC 106266^T^	262	100	7	2,622,354	66.3	BBRG00000000
*D. lutea* NBRC 106155^T^	264	100	40	2,643,741	65.9	BBRC00000000
*D. oxidasica* NBRC 106264^T^	224	85	5	2,623,557	64.1	BBRE00000000
*D. salsinemoris* NBRC 105323^T^	215	67	14	3,209,522	70.2	BBQZ00000000
*D. sediminicola* NBRC 105855^T^	226	92	8	2,460,989	62.7	BBRB00000000

The genome sequences reported in this study will provide a foundation for further phylogenetic, taxonomic, comparative genomic, metagenomic, and functional studies of the genus *Demequina* and related taxa. Recently, we isolated some novel strains which are closely related to the genus *Demequina* and will perform a taxonomic study for the classification of the isolated strains. In the process of the study, the genome sequences presented here will facilitate phylogenetic analyses, including *in silico* DNA-DNA hybridization. Detailed reports of these analyses will be included in a future publication.

### Nucleotide sequence accession numbers. 

The draft genome sequences of *D. aestuarii* NBRC 106260^T^, *D. aurantiaca* NBRC 106265^T^, *D. flava* NBRC 105854^T^, *D. globuliformis* NBRC 106266^T^, *D. lutea* NBRC 106155^T^, *D. oxidasica* NBRC 106264^T^, *D. salsinemoris* NBRC 105323^T^, and *D. sediminicola* NBRC 105855^T^ have been deposited in DDBJ/ENA/GenBank. The accession numbers are listed in Table 1. The versions described in this paper are the first versions.
